# Multiple insecticide resistance in an infected population of the malaria vector *Anopheles funestus* in Benin

**DOI:** 10.1186/s13071-016-1723-y

**Published:** 2016-08-17

**Authors:** Rousseau Djouaka, Jacob M. Riveron, Akadiri Yessoufou, Genevieve Tchigossou, Romaric Akoton, Helen Irving, Innocent Djegbe, Kabirou Moutairou, Razack Adeoti, Manuele Tamò, Victor Manyong, Charles S. Wondji

**Affiliations:** 1 International Institute of Tropical Agriculture, Cotonou, 08 BP 0932 Benin; 2 Liverpool School of Tropical Medicine, Pembroke Place, L3 5QA Liverpool, UK; 3 University of Abomey, Calavi BP 526 Cotonou, Benin; 4 University of Sciences, Arts and Techniques of Natitingou, Ecole Normale Supérieure de Natitingou, Natitingou, BP 123 Benin; 5 International Institute of Tropical Agriculture, Dar Es Salaam, Tanzania

**Keywords:** Malaria, Benin, *Anopheles funestus*, Insecticide resistance, Resistance mechanisms, Malaria vector control

## Abstract

**Background:**

Knowledge on the spread and distribution of insecticide resistance in major malaria vectors such as *Anopheles funestus* is key to implement successful resistance management strategies across Africa. Here, by assessing the susceptibility status of an inland population of *An. funestus* Giles (Kpome) and investigating molecular basis of resistance, we show that multiple resistance and consistent plasmodium infection rate are present in *Anopheles funestus* populations from Kpome.

**Methods:**

The insecticide susceptibility level of collected Anopheles funestus was assessed. Synergist (PBO) was used to screen resistance mechanisms. The TaqMan technique was used for genotyping of insecticide resistant alleles and detecting plasmodium infection levels. The nested PCR was used to further assess the plasmodium infection rate.

**Results:**

The TaqMan analysis of plasmodial infections revealed an infection rate (18.2 %) of *An. funestus* in this locality. The WHO bioassays revealed a multiple phenotypic resistance profile for *An. funestus* in Kpome. This population is highly resistant to pyrethroids (permethrin and deltamethrin), organochlorines (DDT), and carbamates (bendiocarb). A reduced susceptibility was observed with dieldrin. Mortalities did not vary after pre-exposure to PBO for DDT indicating that cytochrome P450s play little role in DDT resistance in Kpome. In contrast, we noticed, a significant increase in mortalities when PBO was combined to permethrin suggesting the direct involvement of P450s in pyrethroid resistance. A high frequency of the L119F-GSTe2 DDT resistance marker was observed in the wild DDT resistant population (9 %RS and 91 %RR) whereas the A296S mutation was detected at a low frequency (1 %RS and 99 %SS).

**Conclusion:**

The presence of multiple resistance in *An. funestus* populations in the inland locality of Kpome is established in this study as recently documented in the costal locality of Pahou. Data from both localities suggest that resistance could be widespread in Benin and this highlights the need for further studies to assess the geographical distribution of insecticide resistance across Benin and neighboring countries as well as a more comprehensive analysis of the resistance mechanisms involved.

**Electronic supplementary material:**

The online version of this article (doi:10.1186/s13071-016-1723-y) contains supplementary material, which is available to authorized users.

## Background

Malaria remains an important health issue in Benin where it is the main cause of morbidity and mortality with children under five and pregnant women being the more vulnerable groups [1]. In this country as across Africa, long lasting insecticide nets (LLINs) and bendiocarb based indoor residual spraying (IRS) are the key tools used for malaria control [1]. In addition to pyrethroids and DDT, organophosphates (Ops) and carbamates (like bendiocarb) are also used to some extent for IRS. However, the success of these control methods is jeopardized by the development by *Anopheles* species of resistance to insecticides such as pyrethroids and DDT which are used for mosquito control. Indeed, resistance against the main insecticides used in public health, such as pyrethroids, carbamates or DDT is increasingly reported in *An. funestus* Giles populations [2–5] from southern Africa (Mozambique [2] and Malawi [6, 7]); East Africa (Uganda) [8, 9]; Central Africa (Cameroon) [2] and West Africa (Ghana, Benin [4, 10]). In Benin, most insecticide susceptibility studies have been conducted on *An. gambiae* Giles with resistance to pyrethroids and DDT recorded in *An. gambiae* (*sensu stricto*) (*s.s*.) and *An. arabiensis* [11–14]. *Anopheles gambiae* (*sensu lato*) (*s.l.*) is a complex of sibling species including two main malaria vectors in sub-Saharan Africa (SSA): *An. gambiae* (*s.s.*) and *An. arabiensis*. As for *An. gambiae* (*s.s.*), two reproductive units previously known as “M” and “S” molecular forms were recently elevated to the species rank: the *An. gambiae* “S” is now designated as *An. gambiae* (*s.s*.) and the *An. gambiae* “M” form is now officially known as *An. coluzzii* [15]. On the other hand, *An. funestus,* the other major malaria vector, has received very little attention. *Anopheles funestus* complex is a group of nine species [16]. Of this complex, *An. funestus* (*s.s.*) is one of the main malaria vectors in the sub-Saharan Africa. Recent studies conducted in the coastal locality of Pahou in Benin revealed the presence of resistance of *An. funestus* to pyrethroids and DDT [4]. Furthermore, Riveron et al. [5] demonstrated that DDT detoxification in *An. funestus* is mainly associated with a single mutation in the GSTe2 gene. Very little is known about the extent and spread of this resistance across Benin notably in the inland localities. Such information can neither be extrapolated from the recorded resistance patterns in coastal regions nor from the neighboring countries as the resistance profiles in *An. funestus* populations vary significantly across Africa. For example the resistance profiles observed in North Cameroon in 2007 (DDT and dieldrin resistance) [3] are different to those observed in southern African countries like Mozambique, Malawi and South Africa for pyrethroid, DDT or carbamate resistance [2, 7, 17, 18]. Pyrethroid resistance has been documented in South Africa [18]. No cases of carbamate resistance have been found in this country. However, carbamate resistance has been well documented in the southern region of Mozambique around the border with South Africa [18]. This resistance pattern in southern Africa is also different to that of East Africa (pyrethroid and DDT resistance but full susceptibility to carbamates) [8] or to that of Ghana (West Africa) (DDT resistance and pyrethroid resistance) [10]. Mechanisms of resistance of *An. funestus* to pyrethroids, carbamates or DDT identified so far are mainly caused by the elevated expression of detoxification genes (metabolic resistance) as neither the L1014F *kdr* mutation nor the G119S *Ace-1* mutation have been detected in this species [4, 8, 19]. However, the detection of A296S *Rdl*^*r*^ mutation in the GABA receptor (involved in dieldrin resistance) of *An. funestus* indicates that target site resistance mechanism is also present in this species [3]. Indeed, P450 genes have been found to be associated with pyrethroid resistance [8, 20, 21] and carbamates resistance as well [22, 23] while mechanisms for the DDT resistance detected in East Africa (Uganda) and West Africa (Benin) are mainly associated to the L119F and GSTe2 mutation [5]. Benin is currently scaling up its malaria control program through Long Lasting Impregnated Nets (LLINs) and IRS [1]. For a successful scaling up of activity, there is a great need to document the susceptibility profiles of malaria vectors to insecticides used in public health and the underlying mechanisms. Generated data will help guiding control programs on the selection of the most suitable insecticides to use and will facilitate the design of country tailored management strategies. However, complementary studies are required to assess the operational impact of the resistance on the efficacy/efficiency of vector control tools against malaria transmission and mortality/morbidity in local populations before giving any clear recommendation to malaria control programs.

In this study, we report the assessment of susceptibility levels to several insecticides used in public health of *An. funestus* population from the locality of Kpome, an inland locality in the southern Benin. We analyzed the infection rate with *Plasmodium* species in this malaria vector population and, investigated the underlying resistance mechanisms. This research will provide current information on the plasmodium infection rate of *An. funestus* and, the insecticide resistance patterns in this species These information will help to improve the control of malaria vectors in Benin.

## Methods

### Area of study and mosquito collection

#### Description of study site

**Fig. 1:**
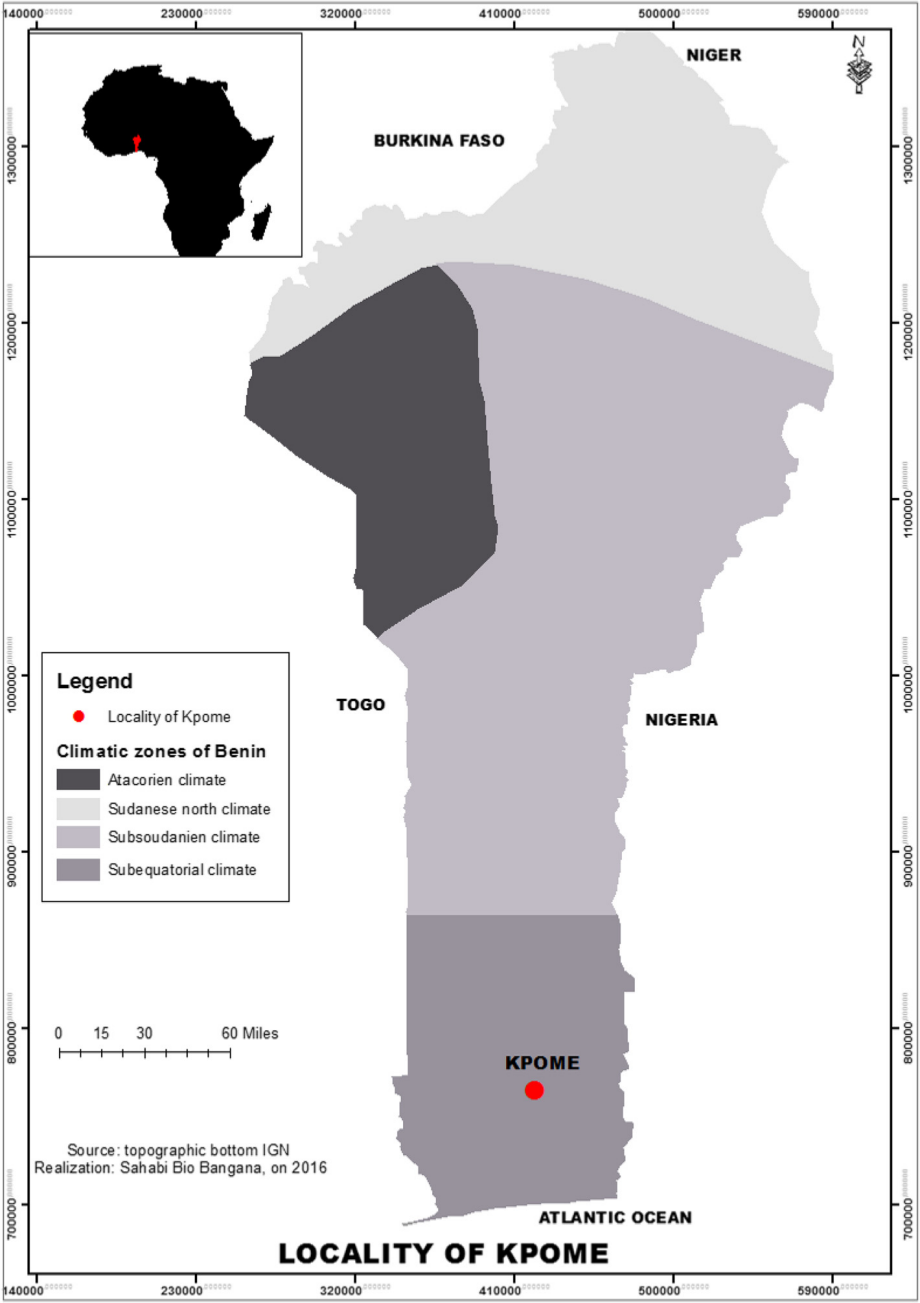
Location of the study site (Kpome) on the map of Benin

Adult *Anopheles* mosquitoes were sampled from the rural locality of Kpome (6°23′N, 2°13′E) in the southern inland region of Benin. Kpome (Fig. 1) is a village constituted of a cluster of traditional houses built with either mud, wooden materials or cement blocks. The average population is 9,000 inhabitants. The main activity here is farming and fishing. The village is surrounded by a river, few swamps and streams which span the various quarters of this locality before joining the river. The vegetation at Kpome is mainly of wet type (aquatic vegetation), shrubs and trees. The constant presence of water bodies around this locality (Kpome) favors the larval development of *An. funestus* mosquitoes

#### Mosquito collection

Electric aspirators and torches were used to collect blood-fed *An. funestus* adult females resting indoors. Collections were conducted in houses between 06:00 and 10:00 am in Kpome. The collection was carried out 2 weeks per month and 30 to 40 rooms were aspirated each sampling day for the period between December 2013 and February 2014. The target sampling period corresponds to the dry season in the southern Benin when *An. funestus* densities are likely to be higher. Blood-fed and gravid females of *An. funetus* (F_0_) were kept in small cups until fully gravid. Eggs were obtained from F_0_ generation using the forced egg laying method [8]. Eggs were sent via courier to the Liverpool School of Tropical Medicine (LSTM) insectary where they were allowed to hatch in small plastic cups with approximately 100 ml of water. L1 larvae emerging from eggs were later transferred into bowls (with 800–1000 ml of mineral water) for rearing and the production of the F1 generation. Larvae rearing was conducted under standard insectary conditions (26 ± 2 °C with a relative humidity of 80 %). The egg batch and emerging larvae from the same female mosquito were reared together and later pooled with larvae from other females if these females were found belonging to the same molecular species (F_0_ females underwent PCR species identification for facilitating larvae pooling). Larvae were fed daily with Tetramin™ baby fish food and the water of each larvae bowl was changed every two days to reduce the mortality rate. The F_1_ adults generated were randomly mixed in cages for subsequent experiments.

### PCR-based species identification

All females used for individual oviposition were morphologically identified as belonging to the *An. funestus* group [24]. Of these females, we subjected a total of 104 (about 75 % of females that oviposited) to DNA extractions using DNeasy extraction kits (Qiagen DNeasy kit) followed by PCR species identification. A PCR was carried out using the protocol described by Koekemoer et al. [25] for molecular identification of collected females. *Anopheles gambiae* collected in the same locality during same period were identified to species and molecular form using the Livak method [26] and the SINE-PCR protocol [27]

### Infection of *An. funestus* with plasmodial species

The plasmodium infection rate was determined using the TaqMan assay described by Bass et al. [28]. The reaction was performed in 10 μl final volume containing 1 × SensiMix (Bioline), 800 nM of each primer described by Bass et al. [28] and 200 nM of each probe labelled with fluorophores, FAM to detect *Plasmodium falciparum*, and HEX to detect *P. ovale*, *P. vivax* and *P. malariae (P. ovm)*. Two *P. falciparum* samples and a mix of *P. ovale, P. vivax* and *P. malariae* were used as positive controls. The real-time PCR MX 3005 (Agilent, Santa) system was used for amplification with the following cycling conditions: 95 °C for 10 min for denaturation followed by 40 cycles of 15 s at 92 °C and 1 min at 60 °C. The same DNA material extracted from the indoor-caught *An. funestus* used for PCR-based species identification was used for the TaqMan *Plasmodium* infection analysis. Additionally, the infection rate of *An. coluzzii* was also estimated for comparison. A nested PCR was performed on all Taqman-*Plasmodium* positive samples to validate results from the TaqMan assay described by [29].

### Insecticide susceptibility assays

Protocols and standard insecticide treated papers supplied by WHO [30] were used to test the susceptibility of *An. funestus* from Kpome to various insecticides. We assessed the susceptibility pattern to six insecticides belonging to the four major public health classes of insecticide: the pyrethroids type I permethrin (0.75 %) and type II deltamethrin (0.05 %); the organochlorines DDT (4 %) and dieldrin (4 %); the organophosphate malathion (5 %) and the carbamate bendiocarb (0.1 %). Knockdown was recorded after 1 h and a 10 % sugar solution was made available to survivors. Final mortality was scored 24 h post-exposure. Because of the absence of susceptible strains of *An. funestus*, the susceptible strain *An. gambiae kisumu* and the wild population of *An. funestus* were exposed to insecticide-treated and non-treated papers as controls [30]. Prior to the experiment, we also confirmed the effectiveness of insecticide-treated papers by exposing the susceptible strain *An. gambiae kisumu* to insecticide-impregnated papers. A mortality in the range 98–100 % indicates susceptibility. A mortality of less than 98 % is suggestive of the existence of resistance and further investigation is needed. If the observed mortality (corrected if necessary) is between 90 and 97 %, the presence of resistant genes in the vector population must be confirmed through additional bioassay tests and/or by conducting molecular assays. If mortality is less than 90 %, confirmation of the existence of resistant genes in the test population with additional bioassays may not be necessary, as long as a minimum of 100 mosquitoes was tested [30].

To further assess the extent of the susceptibility levels to pyrethroids, the F_1_ population from Kpome was further exposed to permethrin for 90 min (30 min additional time). Due to the limited number of females, it was not possible to extend the exposure period for other insecticides. In addition to females, the level of insecticide susceptibility was also assessed in males of *An. funestus*. Each test included control mosquitoes exposed to untreated papers.

### Synergist assay

Following the high level of resistance recorded against 4 % DDT and 0.75 % permethrin, we tested the effect of the synergist PBO in combination with these insecticides to assess the potential role of monooxygenase enzymes. We assessed the synergist PBO because of previous reports of P450s involvement in DDT resistance in *An. gambiae* [31] and pyrethroid resistance in *An. funestus* [32]. Fifty female and 50 male mosquitoes were pre-exposed to 4 % PBO paper for 1 h and immediately exposed to 4 % DDT and 0.75 % permethrin for 1 h. Mortalities following these combined exposures were recorded after 24 h and compared with the results obtained without PBO. Two controls were used for this experiment, one control with mosquitoes exposed to untreated papers and the second control with mosquitoes exposed to the synergist PBO only. Due to the limited number of emerging mosquitoes (F1), we could not test other synergists (e.g. esterase and GST) and other insecticides apart from DDT and permethrin (e.g. deltamethrin, carbamate etc.).

### Genotyping of resistance markers L119F-GSTe2 and A296S-RDL

To assess the role of the L119F-GSTe2 in DDT resistance [5] and the A296S-RDL mutation conferring dieldrin resistance [3, 7], a TaqMan genotyping assay for these markers was used. DNA extracts from adult female *An. funestus* collected indoors (F_0_) were separately genotyped for the GSTe2 and the Rdl^*r*^ allelic variants. In addition, female mosquitoes with known DDT susceptibility phenotypes (F1, dead post-exposure), as defined by the standard WHO protocol [30] were also screened for GSTe2. Taqman SNP genotyping assays were performed in 10 μl volume containing 1× Sensimix (Bioline), 80× primer/probe mix and 1 μl template DNA. Probes were labelled with two specific fluorophores FAM and HEX, FAM to detect the homozygous resistant genotype, HEX to detect the homozygous susceptible genotype and both FAM and HEX to detect the heterozygous genotype. The assay was performed on an Agilent MX3005 real-time PCR machine with cycling conditions of 95 °C for 10 min, followed by 40 cycles at 95 °C for 15 s and 60 °C for 1 min. FAM and HEX fluorescence was captured at the end of each cycle and genotypes called from endpoint fluorescence using the Agilent MXPro software.

### Statistical analysis

Chi-square was used to test for significant differences in the percentage mortalities between female and male mosquitoes exposed to insecticides and Statistica package was used for TaqMan analysis. Genotype frequencies were confirmed to be according to Hardy-Weinberg equilibrium using the Had2know online statistical software (http://www.had2know.com/academics/hardy-weinberg-equilibriumcalculator-2-alleles.html).

## Results

### Species identification and distribution

Results from PCR-based species identification of 104 morphologically identified females of *An. funestus* (*s.l.*) collected from Kpome during the dry season (from December 2013 to February 2014) revealed that they all belong to *An. funestus* (*s.s.*).

### *Plasmodium* infection rates

*An. funestus s.s.* (*s.s*.) from the locality of Kpome were infected (18.27 %) with different species of *Plasmodium* spp. The dominant species of *Plasmodium* recorded in the population was *P. falciparum* (14 infected mosquitoes out of 93 tested) followed by *P. ovale*/ *P. vivax*/ *P. malariae* (*P. ovm*) which were found in two infected mosquitoes. Results from this Taqman analysis also revealed the presence in Kpome of one specimen of *An. funestus* with a mix infection of *P. falciparum* and *P. ovm*. The nested PCR performed on the same specimens revealed 11 mosquitoes infected by *P. falciparum* and one mosquito by *P. malariae.* Using the Taqman analysis, the *An. coluzzii* samples from the same location exhibited infection rate of 13 % (12 out of 93) (Table 1). No significant difference was observed between the infection rates of these two species at Kpome during the survey period (*P* = 0.3070)

**Table 1:** Infectivity of *An. funestus ss* and *An. coluzzii* from Kpome to Plasmodium species (*falciparum, ovale, vivax and malariae)*

Infectivity using the Taqman protocol							Infectivity of *An. funestus* using the nested PCR protocol				
Locality	Species	Tested F_0_	+ve P. fal	+ve P. ovm	+ve P. fal & P. ovm	Total no. of infected *Anopheles* with Taqman (% positivity)	Total no. of infected *An. funestus* using nested PCR (12 out the 17 to Taqman)	+ve P. fal	+ve P. ov	+ve P. viv	+ve P. mal
Kpome	*An. funestus* (*s.s.*)	93	14	2	1	17 ± 7.85 (18)	12	11	0	0	1
Kpome	*An. coluzzii*	93	6	6	0	12 ± 6.83 (13)	–	–	–	–	–

### Susceptibility to insecticides

**Fig. 2:**
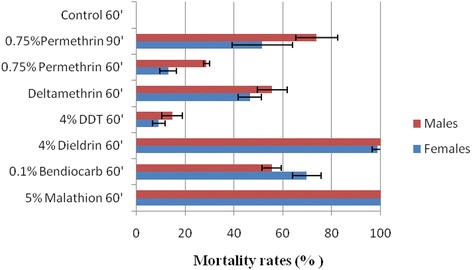
Recorded mortalities following 60 to 90 min exposures of *An. funestus* Kpome to different families of insecticide. Error bars represent 95 % confidence interval

The exposure for one hour of 713 female and male adult *An. funestus* (*s.s.*) from the F_1_ generation to different families of insecticides (pyrethroids, organochlorines and carbamates) revealed significant resistance trends (Fig. 2). High resistance levels were observed against the type I pyrethroid (permethrin) with mortality rates of only 13.0 ± 3 % and 28.7 ± 1 % recorded for females and males, respectively. Even when further exposed for 90 min, low mortality rates were still observed with permethrin with 51.62 % and 73.8 % mortality rates for females and males, respectively. Similarly, a resistance was detected against the pyrethroid type II deltamethrin with mortality rates of 46.5 ± 4.7 % and 55.6 ± 6.2 % for females and males, respectively. The Kpome population was also highly resistant to the organochlorine DDT with mortality rates of 9.1 ± 2.5 % and 14.6 ± 4 % for females and males, respectively. In contrast, for the dieldrin insecticide, a susceptibility profile was observed with mortalities reaching 98.9 ± 2.3 % and 100 % for females and males, respectively. The Kpome population was also resistant to the carbamate bendiocarb with mortality rates of 69.8 ± 5 % and 55.4 ± 3 % for females and males, respectively. However, full susceptibility was observed to the organophosphates malathion with 100 % mortality observed in both males and females (Fig. 2). No significant difference was observed between the mortality of females and males for most tested insecticides (Chi-square test: *χ*^2^ = 9.08, *df*  = 4, *P* = 0.0554)

### Synergist assay with PBO

**Fig. 3:**
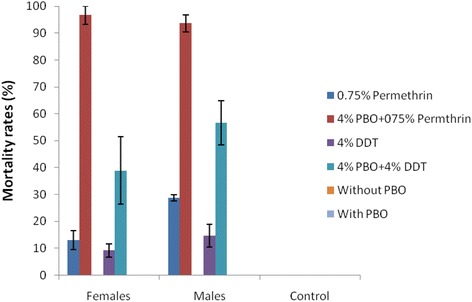
Activities of PBO combined to permethrin and DDT on *An. funestus* (*s.s.*) from Kpome. Error bars represent 95 % confidence interval

Due to the high resistance observed against permethrin and DDT in Kpome, a synergist assay with 4 % PBO was carried out to assess the potential role played by cytochrome P450 genes in observed resistance. For DDT combined with PBO, mortality rates increased from 9.2 to 38.9 % for females and from 14.7 % to 56.6 % (Fig. 3) for males, suggesting that other enzymes such as glutathion-S-transferase could play an important role in DDT resistance. In contrast, a high recovery of susceptibility was observed to permethrin after pre-exposure to PBO (PBO + permethrin) with a mortality rate raising from 13.0 to 96.7 % for females and 28.7 % to 93.6 % for males suggesting a major role of cytochrome P450s and oxidases in the observed pyrethroid resistance (Fig. 3). No mortality was recorded in control tubes.

### Genotyping of the GSTe2 alleles in the *An. funestus* population of Kpome

Following the observed high resistance profile of *An. funestus* Kpome to DDT, we screened for the GSTe2 mutation which was recently shown to confer DDT resistance in the coastal *An. funestus* population from Pahou in the Benin [5]. Results showed a high presence of the L119F-GSTe2 mutation in the wild *An. funestus* population from Kpome with 91 % individuals homozygote for the 119 F resistant allele (84 out of 92 individuals tested) whereas 9 % were RS. The recorded genotypic frequency was found at Hardy-Weinberg (HW) equilibrium with a *P*-value of 0.6628 and the expected HW frequencies of 84.17 RR, 7.65 RS and 0.17 SS. No SS individual was recorded in Kpome showing that the GSTe2 is nearing fixation in the *An. funestus* population of Kpome as previously observed in Pahou [5] (Additional file 1: Figure S1).

### Association of the L119F-GSTe2 mutation with DDT resistance

**Fig. 4:**
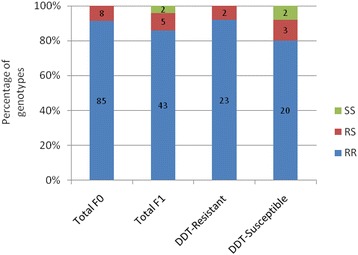
Proportions of GSTe2 genotypes sorted in the wild Kpome *An. funestus* population (F_0_) and, the DDT-exposed individuals (F_1_; alive and dead individuals post-DDT exposure)

Genotyping of the L119F-GSTe2 mutation in 25 DDT resistant (alive post-exposure) and 25 DDT susceptible (dead post-exposure) revealed high frequencies of the 119 F resistant allele in both resistant and susceptible mosquitoes (Fig. 4). A higher frequency of homozygote 119 F resistant allele was observed in the resistant sample at 92 % (23 RR/RS out of 25 alive mosquitoes screened) *vs* 80 % (20 RR/RS out of 25 dead mosquitoes screened) in susceptible. The *P*-value (*P* = 0.2274) did not reveal any statistical difference of the resistant alleles in both alive and dead mosquitoes showing that the L119F-Gste2 mutation is almost fixed in Kpome population and therefore, mutations on Gste2 cannot be used for discriminating DDT dead and alive phenotypes in such localities where the mutation is almost fixed. To obtain the 25 dead *An. funestus* used in this experiment, we conducted several additional bio-assays with DDT.

### Genotyping of the A296S Rdl alleles in Kpome

Despite the relative susceptibility of the Kpome population to dieldrin, we genotyped the A296S mutation to confirm whether its frequency correlates with observed susceptibility in Kpome. Results showed a very low presence of Rdl^*r*^ in the genotyped F_0_ population of Kpome; out of 92 individuals genotyped, 91 were SS (99 %) and only one was RS (1 %). No individual was recorded with RR mutation showing that the selection mechanism for this mutation in wild *An. funestus* populations of Kpome is still very low (Additional file 2: Figure S2). This low allelic frequency of Rdl^*r*^ resistant allele in the population further correlates with the high susceptiblity of the *An. funestus* (*s.s.*) in Kpome to dieldrin (Fig. 2). The genotypic frequency of Rdl was found at Hardy-Weinberg equilibrum with a *P*-value of 0.9582 and the expected frequencies of 0 RR, 0.99 RS and 91 SS.

## Discussion

The susceptibility profile analysis of an inland population of *An. funestus* has revealed for the first time in Benin that multiple resistance patterns extent beyond the coast; the Kpome population having demonstrated resistance to several insecticide classes in our study.

### Contribution of *An. funestus* to malaria transmission

This study further confirms the presence of *An. funestus* in the southern Benin as recently published by Djouaka et al. [4] and highlights its consistent infection level with *Plasmodium* species. The Kpome *An. funestus* population was found with consistent *Plasmodium* infections during the dry season (infection rate of 18.2 %). It is worth mentioning that the analysis of *Plasmodium* spp. in *An. funestus* also revealed that some mosquitoes could be co-infected with more than two species, namely *P. falciparum* and any of the *P. ovale, P. malaria* or *P*. *vivax*. When the infection rate of *An. funestus* was compared with that of *An. coluzzii* mosquitoes collected in Kpome during a similar period (dry season), we noticed a relatively high infection rate of indoor-collected *An. funestus* compared to indoor-collected *An. coluzzii*. This comparative analysis shows the high competitiveness of *An. funestus* in the transmission of malaria in southern Benin. The infection rates (infection rates > 15 %) recorded in both species (*An. funestus* and *An. coluzzii*) during the same period predicts possibilities of high malaria transmission and incidence in this locality during dry seasons. When similar samples were analyzed with the nested PCR, a relatively low number of infected mosquitoes was recorded; this certainly reveals a higher sensitivity of the Taqman technique compared to the nested PCR technique [28]. The infection rate recorded in this *An. funestus* population is as high as previously recorded in other localities across Africa. For example, parasite rates of 22 % [33] and 27 % [34] have been recorded for this species in the past in South Africa and more recently, in West Africa, mean rates of infectivity between 3 % and 15 % have been observed in Burkina Faso [35, 36]. During the same studies, Costantini et al. [35] recorded up to 50 % *An. funestus* in one village in November 1991 (*n* = 56) positive for *P. falciparum* circumsporozoite and Dabire et al. [36] recorded up to 20 % of infected *An. funestus* in Lena in August 2000 (*n* = 40) using the same Elisa method. In southern Benin, a recent study using multiplex real-time PCR assays revealed *P. falciparum* infection and mixed infections with *P. malariae* and/or *P. ovale* in 13.6 % of *An. funestus* collected [37]. Overall, high infection rates of *An. funestus* observed in some villages in West Africa in particular at the end of the rainy season and its related high anthropophily, contribute to the main role of this species in malaria transmission, surpassing in some cases other main vectors like *An. gambiae * (s.s.). The implication of *An. funestus* in malaria transmission during the dry season was also documented in Ghana [38], Nigeria [39], Burkina Faso [35, 36] and more recently, in southern Benin [40]. No *Plasmodium vivax* was found in analysed mosquitoes from Benin. The infection rate recorded with the Taqman technique was higher than that obtained with the nested PCR; this may be due to the higher sensitivity of the Taqman assay compared to the nested PCR technique [28].

### Multiple resistance could be widespread in *An. funestus* in Benin

The multiple resistance pattern of the *An. funestus* population in Kpome is similar to that of the coastal location of Pahou [4] suggesting that multiple resistance could be widespread in *An. funestus* populations in Benin at least in the southern region. This multiple resistance concerns several insecticide classes including the organochlorine DDT, pyrethroids and carbamates. The *An. funestus* population is highly resistant to both type I and II pyrethroids. The level of this resistance is consistently higher than that reported back in 2011 for the coastal population of Pahou. For example for females, the mortality rate after permethrin exposure was 66.7 % in Pahou in 2011 but only 13 % in Kpome in 2014. Similarly, for the type II pyrethroid, deltamethrin, a higher mortality rate of 88.6 % was observed in Pahou compared to only 46.5 % in Kpome. This difference could suggest an increase in the overall level of pyrethroid resistance in Benin in three years. This increase could be related to the increased ITNs coverage across Benin and additional selection factors such as agricultural use of pesticides [41, 42], and spilled petroleum products [43]. High resistance was recently reported for this species in Malawi where a significant rise of levels of pyrethroid resistance was detected when comparing a 2014 population of southern Malawi to the 2009 population from the same location [7]. Same increase was also reported in Uganda for *An. funestus* [9] suggesting that pyrethroid resistance is being further selected in field populations and this is a concern for the continued effectiveness of pyrethroid-based interventions such as LLINs. This concern is further compounded by the increase in  resistance also observed in *An. gambiae* (*s.l.*) populations from Benin [14, 44], Burkina Faso and Cameroon [45–47].

The resistance recorded in Kpome is higher against permethrin (type I pyrethroid) than to deltamethrin (type II pyrethroid). This resistance pattern is different to that observed in southern or eastern Africa where resistance to type II pyrethroid is higher than to type I [6, 9, 19, 48]. This difference may underline the existence of some differences in the resistance mechanism against pyrethroids in Benin compared to the southern and Eastern Africa regions. This could be explained by the significant association established between the L119F-GSTe2 mutation and permethrin resistance but not for deltamethrin [21]. Moderate permethrin resistance was also detected in other West African *An. funestus* populations from Obuasi in Ghana [10] but no pyrethroid resistance was reported in *An. funestus* from Soumossou in Burkina Faso in 2007, although it may have changed by now [36] indicating that this resistance may not yet be widely distributed across West Africa. This resistance to pyrethroids is of great concern for malaria control programs and, there is a risk that if such resistance is not managed properly, it can be further selected by ongoing control interventions such as the pyrethroid impregnated LLINs to a level that will seriously impact the success of future control programs. The current trend of pyrethroid resistance recorded with the main malaria vectors (*An. gambiae* and *An. funestus*) in Benin [4, 13, 14, 49] is a concern for the success of malaria control program focussed on pyrethroid-based LLINs. Beyond the evaluation of pyrethroid resistance profile of malaria vectors, a pilot Phase II entomological study examining the effectiveness of using ITNs at two sites in Benin has already given clear evidence of pyrethroid-based ITNs failing to control a pyrethroid-resistant *An. gambiae* population [50]. Nevertheless, at the epidemiological level, a recent study performed in the South Benin revealed that the observed pyrethroid resistance of malaria vectors seems to date not to have affected the impact of LLINs; therefore, the use of LLINs remains associated with reduced malaria prevalence irrespective of resistance [51]. The DDT resistance level observed in this population is one of the highest in Africa after the one documented at Pahou where no mortality was recorded after 1 h exposure [4]. This confirms that DDT resistance level is high in Benin in contrast to southern Africa where such resistance has just been reported in Zambia and Malawi [7, 22]. The level of DDT resistance in Benin is also higher than the level observed in East Africa where mortality rates are above 40 % [9, 48]. The presence of DDT resistance within Benin is certainly going to reduce the options available for insecticide resistance management of *An. funestus* populations particularly with the observed resistance to pyrethroids and carbamates. This multi-resistance of *An. funestus* species to several groups of insecticides brings more complexities in the control of malaria as this high DDT resistance is also prevalent in several populations of *An. gambiae* in Benin [4, 49]. The presence of insecticide-resistant populations of *An. funestus* in costal urbanized and polluted localities (e.g. Pahou) and in the inlands (e.g. Kpome) with less urbanization and less pollution could be associated with gene flow among *An. funestus* populations in southern Benin. In addition, exogenous factors such as agriculture, urbanization (pollution including petroleum products) and ITNs/IRS (the use of public health insecticides) might have also contributed to the observed high resistance profile of mosquitoes [4, 11, 43, 52–54]. In the coastal localities of Benin all exogenous factors (ITNs/IRS, urban agriculture with high pesticide utilization, rapid urbanization/urban pollution and spilled petroleum products from vehicles, repair shops and road-side re-sellers) simultaneously act on mosquito population and intensely select for insecticide resistance, whereas in the inland localities the main dominant factor for selection of DDT resistance is the agricultural use of pesticides. *An. funestus* population from Kpome is also resistant to the carbamate bendiocarb. This observed bendiocarb resistance at Kpome confirms the presence of bendiocarb resistance in *An. funestus* in the southern Benin as earlier reported by Djouaka et al. [4] in Pahou. Also, the bendiocarb resistance was recently reported in *An. gambiae* (*s.s.*) in Atacora, in the northern part of Benin [55]. This bendiocarb resistance recorded in this study raises a special concern for National Malaria Control programs which, because of high resistance to pyrethroids and DDT, are currently introducing bendiocarb based IRS for malaria vector control in West African countries [56]. Such resistance was observed in *An. gambiae* population in Benin three years after the large implementation of bendiocarb based IRS [57]. The rapid selection of bendiocarb resistance in *An. gambiae* was also observed in other West African countries, e.g. Burkina Faso following the introduction of IRS by the US PMI program [58]. The same process probably affected *An. funestus* considering its high endophilic behavior. The full susceptibility to the organophosphate malathion is similar to results throughout Africa showing that *An. funestus* populations remain susceptible to this insecticide class which can therefore be used as alternative insecticide for resistance management strategies. However, the toxicity of malathion might limit its adoption by malaria vector control programs. Recently, other less toxic OP candidates like pirimiphos methyl [59] or products belonging to other classes of insecticide like the pyrrole insecticide chlorfenapyr [60] and the indoxacarb oxadiazine [61] revealed promising in Phase II studies against pyrethroid-resistant malaria vectors in Benin and elsewhere in sub-Saharan Africa.

The nearly full susceptibility to dieldrin correlated very well to the extremely low frequency of the Rdl^*r*^ mutation in is similar to previous observation in Pahou [8] but contrasts to the high frequency observed in neighboring Burkina Faso (Vallée du Kou) [46] and Cameroon [7]. In Burkina Faso, insecticide susceptibility tests performed in 1967 in the Soumousso village already reported resistance to dieldrin in *An. funestus*, [62] highlighting that insecticide resistance selected in the past could persist nowadays even in the absence of selection pressure (i.e. contemporary use of the same or related insecticide) in particular for resistance mechanisms that have no fitness cost for the insect.

### Analysis of underlying mechanisms of the observed multiple resistance profiles

The underlying mechanisms of the multiple resistance pattern observed in this population was explored through various means. The synergist assay with PBO, an inhibitor of cytochrome P450 monooxygenases, indicated that this enzyme family does not play a major role in the high DDT resistance observed in Kpome. However, when mosquitoes where pre-exposed to PBO before being exposed to permethrin (PBO + permethrin), mortalities increased about 9-fold almost reaching 100 % for both males and females, showing that P450 monooxygenases are playing a major role in pyrethroid resistance at Kpome. This also further supports that pyrethroid resistance is mainly driven by metabolic resistance in field populations of *An. funestus* as observed throughout Africa with little evidence of knockdown resistance [19, 21]. In this study, because of the low number of mosquitoes collected, we did not carry out the biochemical analysis to determine levels/activities of known detoxifying enzyme systems including monooxygenases, non-specific esterases and GSTs as well as to detect the presence of an altered acetylcholinesterase associated with carbamate resistance. However, biochemical analysis performed on wild *An. funestus* from Ghana suggested that DDT and pyrethroid resistance may be metabolically mediated, although there were no clear correlations between enzyme levels/activities and insecticide resistance across families. Furthermore, an altered acetylcholinesterase conferring carbamate resistance was evidenced [10]. The recovery of susceptibility to pyrethroids after PBO exposure is in line with the absence of any kdr mutation in *Anopheles funestus* as previously reported in the Pahou population [4]. The very high frequency of the 119 F-GSTe2 resistant allele in Kpome, is similar to that observed in Pahou in 2011. Both data from Pahou and Kpome correlate the observed high level of DDT resistance in both localities and suggest that this mutation might play an important role in DDT resistance in West Africa. The results observed in Kpome are also in line with the detection of high frequency of this resistance allele in other DDT-resistant populations in West Africa notably in Cameroon (48.2 %), Ghana (44.2 %) and Burkina Faso (25 %), in accordance with the previously reported prevalence of DDT resistance in these countries [5]. The resistant 119 F allele has also been detected in the eastern African countries of Uganda (20.4 %) and Kenya (7.8 %) but with lower frequencies, reflecting the moderate level of DDT resistance that was previously reported [8, 9]. However the above observations are in contrast with the complete absence of the 119 F in southern African populations despite the recent detection of DDT resistance in that region suggesting the presence of different mechanisms of DDT resistance in *An. funestus* [2, 7]. The observed high frequency of 119 F in both DDT susceptible and resistant phenotypes shows that this mutation is near fixation at Kpome; therefore, bioassays with DDT cannot be used for discriminating mutant individuals in this population. Such observations have also been documented with the *kdr* mutation in *Anopheles gambiae* in areas of high pyrethroid resistance and where this mutation *(kdr)* is almost fixed. We noticed in such areas that some dead mosquitoes post-pyrethroid bio-assays were carrying the *kdr* mutation [63, 64]. The 119 F resistant allele in Kpome was probably selected during large use of DDT for agriculture (i.e. against pests) [41, 42], and/or public health (against vectors) [54]. Another possibility is that the 119 F allele could also be selected not by DDT but by pyrethroid-based interventions as it was shown that GSTe2 can metabolize permethrin [5].

## Conclusion

This study has provided relevant information on the *Plasmodium* infection rate in *An. funestus* (*s.s.*) in the southern Benin. The study also highlighted the multiple resistance profile of *An. funestus* (*s.s.*) to insecticides used in public health such as permethrin, deltamethrin, DDT and bendiocarb. This multiple resistance profile previously observed in the Pahou population and now in the Kpome population highlights the need for further studies to assess the extent and geographical distribution of these resistances in *An. funestus* populations throughout Benin and West Africa. At the same time, these results call for the development of a more comprehensive analysis of the resistance mechanisms involved in order to improve the implementation and management of future control programs against this species and other *Anopheles* mosquitoes locally involved in malaria transmission.

## Abbreviations

DDT, dichlorodiphenyltrichloroethane; EST, esterase; WHO, World Health Organization

## Additional files

Additional file 1: Figure S1. TaqMan screening of the GSTe2 genotypes in wild *An. funestus* (*s.s.*) from Kpome showing a high presence of RR individuals and the absence of SS individuals. (DOC 81 kb) Additional file 2: Figure S2. TaqMan screening of the Rdlr genotypes in wild *An. funestus* (*s.s.*) from Kpome showing a high presence of SS individuals and a very low frequency of SS individuals. (DOC 79 kb)
