# Supplementary material for: Multiple insecticide resistance in an infected population of the malaria vector Anopheles funestus in Benin
Source: Parasit Vectors. 2016 Aug 17;9:453. doi: 10.1186/s13071-016-1723-y (PMC4987972; doi:10.1186/s13071-016-1723-y)
Supplement: Additional file 2: Figure S2. — TaqMan screening of the Rdlr genotypes in wild An. funestus (s.s.) from Kpome showing a high presence of SS individuals and a very low frequency of SS individuals. (DOC 79 kb) [file 13071_2016_1723_MOESM2_ESM.doc]

**Additional file 2**

**Figure S2:** TaqMan screening of the Rdl*^r^* genotypes in wild *An. funestus ss.* from Kpome showing a high presence of SS individuals and a very low frequency of SS individuals.
